# Utility of Point-of-Care Ultrasound for the Rapid Evaluation of Acute Sialadenitis: A Case Report

**DOI:** 10.7759/cureus.24881

**Published:** 2022-05-10

**Authors:** Jeffrey A Kramer, Roxanna Pourmirzaie

**Affiliations:** 1 Emergency Medicine, University of Pennsylvania, Philadelphia, USA; 2 Hospital Medicine, University of Pennsylvania, Philadelphia, USA

**Keywords:** ultrasound, submandibular gland, ultrasound (u/s), emergency medicine, point-of-care ultrasound (pocus), point-of-care-ultrasound, sialadenitis

## Abstract

Acute suppurative sialadenitis is a bacterial infection of the salivary glands which leads to a painful, tender, and swollen salivary gland. Point-of-care ultrasound (POCUS) is a limited ultrasound performed by the provider to answer a specific clinical question. We present a case describing the efficient utilization of POCUS for the rapid diagnosis of acute suppurative sialadenitis in the Emergency Department.

## Introduction

Acute suppurative sialadenitis is a bacterial infection of the salivary glands. It typically affects one gland at a time and is thought to be due to the impaired flow of saliva within the salivary ducts. In this low-flow state, oral bacteria can ascend the duct and initiate infection. *Staphylococcus aureus* is the most common bacteria cultured in these infections. Risk factors for developing this infection include sialolithiasis, dehydration, immunocompromise, ductal stenosis, and Sjögren syndrome [1,2].

Point-of-care ultrasound (POCUS) was adopted by emergency medicine (EM) over three decades ago and is a requirement in EM residency training. POCUS fellowships can be accredited, and fellowship-trained individuals may seek certification by the American Board of Emergency Medicine [3]. Here, we present the case of a woman presenting with painful neck swelling and purulent intraoral drainage who was diagnosed with sialadenitis by POCUS in the Emergency Department (ED).

## Case presentation

**Figure 1:**
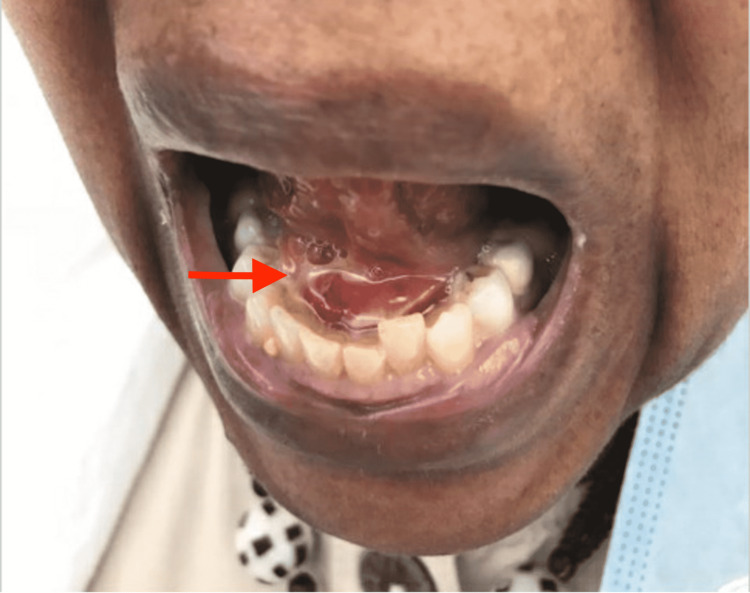
Purulent drainage from right-sided Wharton’s duct (see arrow).

A 72-year-old female with a medical history of hypertension and hyperlipidemia presented to the ED with a three-day history of right face and neck swelling, intraoral pain, and purulent drainage. She did not complain of fever, cough, or chills. The patient was otherwise healthy. Vital signs were notable for a blood pressure of 160/97 mmHg, a pulse of 85 beats per minute, the temperature of 37.7°C (99.8°F), and a respiratory rate of 16 breaths per minute. Labs were notable for a white blood cell count of 10.1 K/µL, but otherwise unremarkable. Physical examination was notable for a slightly swollen right face, with overt purulent drainage from the right submandibular duct or Wharton’s duct (Figure 1).

The tongue was not enlarged, and the airway was patent. The lungs were clear to auscultation with no associated respiratory distress. In the ED, a POCUS was completed, in addition to a computed tomography (CT) scan. Ultimately, Ear, Nose, Throat (ENT) was consulted.

**Figure 2:**
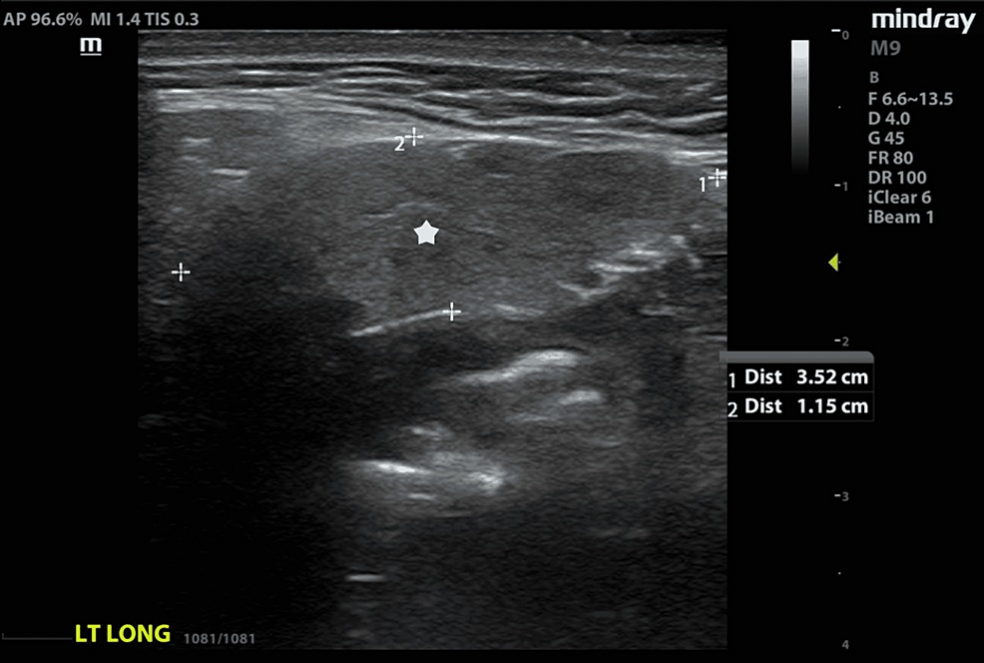
Unaffected left submandibular gland with homogeneous echotexture and no visible Wharton’s duct (see star).

**Figure 3:**
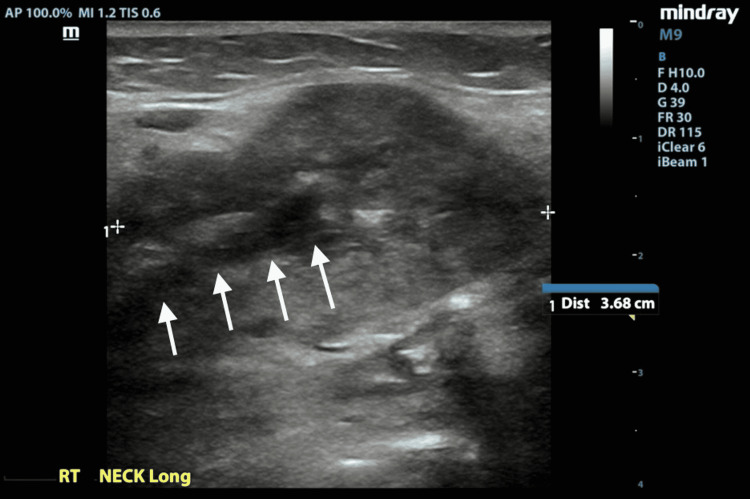
POCUS of the right submandibular gland: longitudinal view of the enlarged right submandibular gland with heterogeneous echotexture and dilated Wharton’s duct (see arrows). POCUS: point-of-care ultrasound (POCUS)

**Figure 4:**
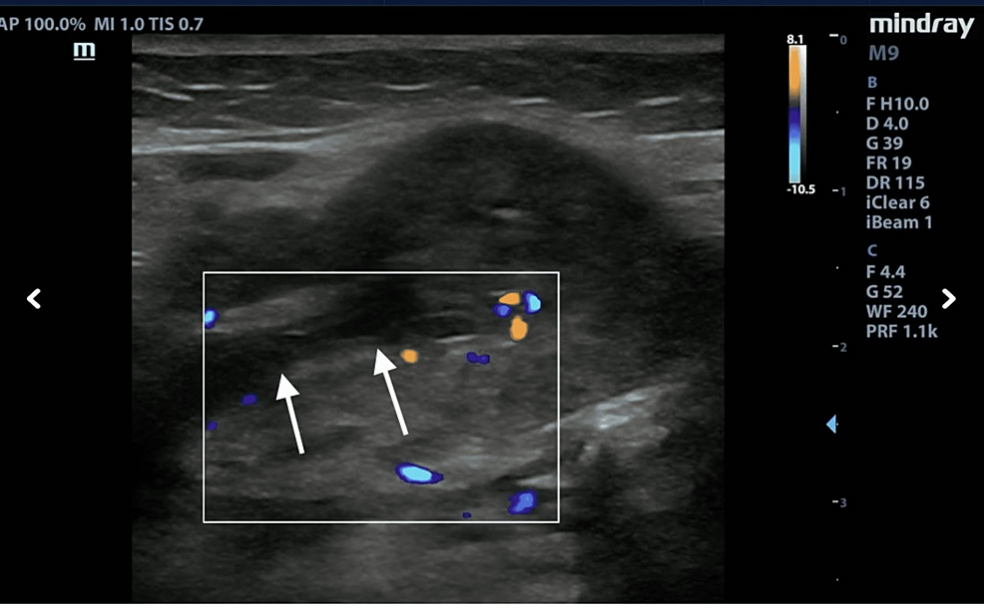
Color Doppler differentiating Wharton’s duct from vascular structures (see arrows).

A Mindray M9 ultrasound was used with a linear, high-frequency probe set to 10 MHz. The scan was performed by an emergency ultrasound fellow under the direction of an experienced EM ultrasound faculty. The ultrasound was performed with the patient in a supine position, with the neck extended and rotated away from the side being examined. The submandibular gland is best seen in an oblique plane and was scanned as such [4]. The glands should be examined in both the transverse and longitudinal planes. Color Doppler was utilized to differentiate vascular structures within and surrounding the gland. The normal submandibular gland displayed a homogenous echotexture (Figure 2), while the affected gland displayed a heterogenous echotexture with internal hypoechoic and hyperechoic areas. Wharton’s duct was visible in the affected gland (Figures 3, 4).

**Figure 5:**
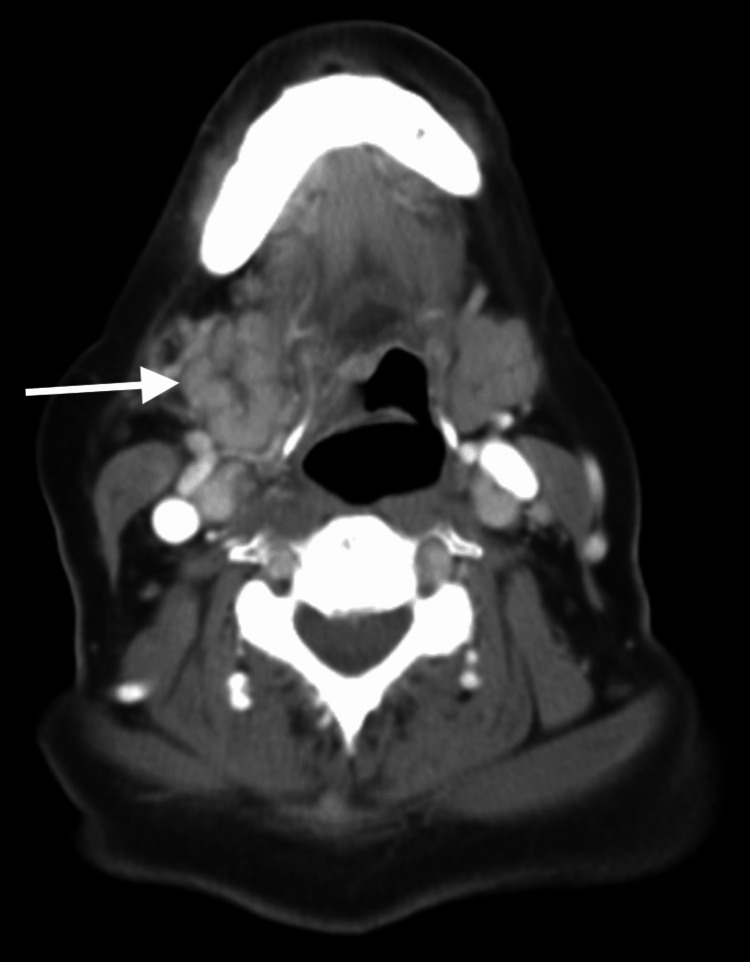
CT of the neck with IV contrast: acute sialadenitis of the right submandibular gland with mildly dilated intra and extraglandular inflamed ducts without visualization of radiopaque calculi (see arrow). CT: computed tomography; IV: intravenous

Wharton’s duct is typically only visible if dilated [5,6]. The affected submandibular gland was enlarged and measured 37 mm × 25 mm, while the unaffected gland measured 35 mm × 12 mm. There was no evidence of sialolithiasis. The patient underwent a CT scan with contrast (Figure 5).

CT showed acute sialadenitis of the right submandibular gland with mildly dilated intra and extraglandular inflamed ducts without visualization of radiopaque calculi, which corroborated the POCUS findings. She was discharged home safely with a course of antibiotics, steroids, sialogogues, and instructions for ENT follow-up. On outpatient follow-up nine days later, her symptoms had resolved.

## Discussion

Acute suppurative sialadenitis is a bacterial infection of the salivary gland, most commonly caused by *Staphylococcus aureus*. Other pathogens include *Haemophilus influenzae*, viridans streptococci, *Streptococcus pneumoniae*, and *S. pyogenes*. *Escherichia coli* and *Pseudomonas aeruginosa* can be found in cases of nosocomial infection [3,7]. A decrease in salivary flow allows the bacteria to ascend into the gland and is a primary factor in this condition [1,2]. Conditions that slow the flow of saliva include sialoliths, dehydration, ductal stenosis, dental infection with subsequent swelling, and tumors. Autoimmune disorders such as sarcoidosis and Sjögren’s syndrome lead to decreased production of saliva and predispose to infection. Immunosuppression is also a risk factor. This is especially true in patients receiving chemotherapy who are also often dehydrated [2]. Numerous medications, including diuretics, antipsychotics, and opiates, can slow saliva production and flow and may play a role in the development of this infection [2].

Acute suppurative sialadenitis presents with rapid onset of painful swelling of the affected salivary gland. Fever may be present. Examination typically reveals an enlarged, tender salivary gland which may be erythematous. An intraoral examination often demonstrates dry mucus membranes and purulent drainage from the salivary duct, as seen in this case [2,8].

Treatment consists of hydration, improved oral hygiene, antibiotics, and, in cases of sialolithiasis or ductal stenosis, removal of the obstruction. Antisialagogic medications should be stopped. Antimicrobial therapy should broadly cover aerobic and anaerobic pathogens using agents such as amoxicillin/clavulanate or clindamycin [2,7]. In cases of extensive soft-tissue swelling, corticosteroid therapy should be considered [8]. In cases where symptoms fail to improve with antibiotics, the possibility of a salivary gland abscess should be considered [2].

CT is traditionally the imaging modality of choice and is considered the gold standard for the detection of inflammatory diseases of salivary glands [5]. In our case, POCUS with a linear, high-frequency probe revealed a large submandibular gland, measuring approximately 37 mm × 25 mm with a heterogeneous echotexture. For reference, the average length of the normal submandibular salivary gland in females is approximately 33 mm, while the average width is approximately 13 mm [9]. An enlarged Wharton’s duct was also noted. Wharton’s duct is typically not visible under normal circumstances [5,6]. No sialoliths were visualized on the POCUS, which are typically hyperechoic bodies with acoustic shadowing [6].

Although the modality of choice, CT has many drawbacks when compared to POCUS. CT takes longer to obtain, requires the patient to be transported to the CT scanner, exposes the patient to ionizing radiation, and requires intravenous (IV) access for the administration of contrast. IV contrast poses risks to the patient such as allergic reaction, anaphylaxis, and contrast extravasation. In this case, the CT took three hours and 18 minutes from order to the final read. The POCUS was requested by the primary provider and was completed 10 minutes later. Ultrasound is widely available and a useful tool, particularly in remote or underserved areas, and as shown in this case, rapidly increases the time to diagnosis compared to imaging with CT.

## Conclusions

This case highlights the utility of POCUS in obtaining a rapid diagnosis in patients with salivary gland disease. In addition to its temporal benefits, POCUS does not expose patients to ionizing radiation, require IV access, or necessitate transport out of the ED to the CT scanner. Ultimately, we believe POCUS is a highly useful and underutilized tool in the evaluation and diagnosis of acute inflammatory conditions of the salivary glands.
